# [*N*,*N*′-Bis(2,6-diisopropyl­phen­yl)pentane-2,4-diamine­(1–)-2κ^2^ *N*,*N*′]-μ_2_-chlorido-1:2κ^2^ *Cl*:*Cl*-chlorido-2κ*Cl*-bis­(1,2-di­methoxy­ethane-1κ^2^ *O*,*O*′)iron(II)lithium

**DOI:** 10.1107/S1600536810018222

**Published:** 2010-05-26

**Authors:** Rafał Grubba, Łukasz Ponikiewski, Łukasz Tomorowicz, Jerzy Pikies

**Affiliations:** a Chemical Faculty, Gdansk University of Technology, Narutowicza 11/12, Gdansk PL-80233, Poland

## Abstract

In the title compound, [FeLi(C_29_H_41_N_2_)Cl_2_(C_4_H_10_O_2_)_2_], the Fe^II^ atom is coordinated by two N and two Cl atoms, generating a distorted FeN_2_Cl_2_ tetra­hedral geometry. Additionally, one of the chloride atoms bridges to a lithium ion, which is solvated by two dimethoxy­ethane mol­ecules and is coordinated in a distorted trigonal-bipyramidal environment. The central Fe, Cl (× 2) and Li atoms are coplanar with a maximum deviation of 0.034 Å.

## Related literature

For the crystal structure of the 2,4-bis­(2,6-diisopropyl­phenyl­imido)pentane ligand, see: Smith *et al.* (2001 ▶); Evans *et al.* (2003 ▶). For a related iron(II) structure, see: Sciarone *et al.* (2006 ▶). For details of the preparation, see: Kovacs *et al.* (1996 ▶); Panda *et al.* (2002 ▶). For related syntheses, see: Baum *et al.* (2004 ▶).
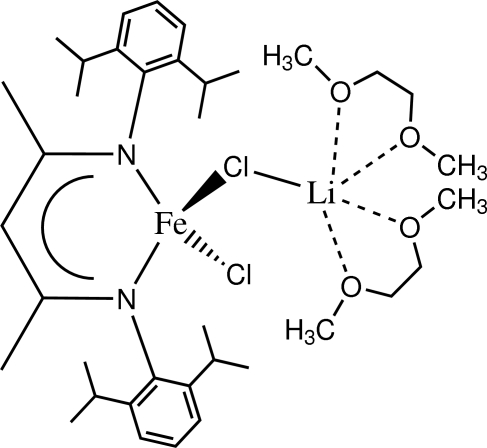


## Experimental

### 

#### Crystal data

[FeLi(C_29_H_41_N_2_)Cl_2_(C_4_H_10_O_2_)_2_]
*M* *_r_* = 731.57
Monoclinic, 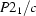

*a* = 10.1467 (5) Å
*b* = 19.8186 (10) Å
*c* = 20.6289 (11) Å
β = 104.962 (4)°
*V* = 4007.7 (4) Å^3^
*Z* = 4
Mo *K*α radiation
μ = 0.55 mm^−1^
*T* = 150 K
0.27 × 0.24 × 0.13 mm

#### Data collection

Oxford Diffraction Xcalibur Sapphire2 diffractometer
Absorption correction: multi-scan (*CrysAlis PRO*; Oxford Diffraction, 2009 ▶) *T*_min_ = 0.983, *T*_max_ = 1
23717 measured reflections
7045 independent reflections
4320 reflections with *I* > 2σ(*I*)
*R*_int_ = 0.042

#### Refinement

*R*[*F* ^2^ > 2σ(*F* ^2^)] = 0.041
*wR*(*F* ^2^) = 0.108
*S* = 0.89
7045 reflections
438 parameters
H-atom parameters constrained
Δρ_max_ = 1.63 e Å^−3^
Δρ_min_ = −0.29 e Å^−3^

### 

Data collection: *X-AREA* (Stoe & Cie, 1997 ▶); cell refinement: *X-AREA*; data reduction: *X-RED*; program(s) used to solve structure: *SHELXS97* (Sheldrick, 2008 ▶); program(s) used to refine structure: *SHELXL97* (Sheldrick, 2008 ▶); molecular graphics: *ORTEP-3* (Farrugia, 1997 ▶); software used to prepare material for publication: *WinGX32* (Farrugia, 1999 ▶).

## Supplementary Material

Crystal structure: contains datablocks I, global. DOI: 10.1107/S1600536810018222/kp2260sup1.cif

Structure factors: contains datablocks I. DOI: 10.1107/S1600536810018222/kp2260Isup2.hkl

Additional supplementary materials:  crystallographic information; 3D view; checkCIF report

## Figures and Tables

**Table 1:** Selected bond lengths (Å)

Fe1—N1	2.020 (2)
Fe1—N2	2.029 (2)
Fe1—Cl1	2.2982 (8)
Fe1—Cl2	2.3207 (7)
Cl2—Li1	2.463 (4)
O1—Li1	2.058 (5)
O2—Li1	2.088 (5)
O3—Li1	2.081 (5)
O4—Li1	2.126 (5)

**Fig. 1.**
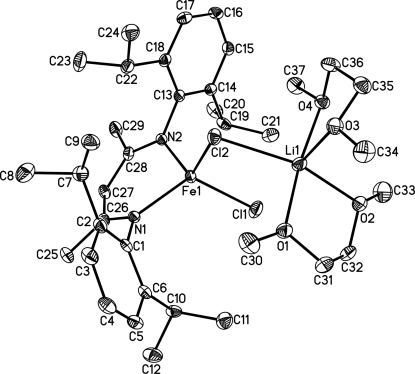
The molecular structure of the title molecule with the atom-numbering. Displacement ellipsoids are drawn at the 30% probability level.

## supplementary crystallographic information

### Comment

In the course of our studies on phosphorus-iron chemistry, we have syntesised the title complex of iron(II) [LFeCl{*µ*-Cl}Li(DME)_2_] (L = [{(2,6-^i^Pr_2_H_3_C_6_)NC(CH_3_)}_2_CH]^-^) (1). This compound turned to be the main product in reaction ^t^Bu_2_P—P(SiMe_3_)Li^.^2THF with [LFeCl_2_] (L = {(2,6-^i^Pr_2_H_3_C_6_)N(CH_3_)C}_2_CH]- (molar ratio 2:1 in DME). We observed reduction of starting complex of iron(III) to iron(II) by lithium salt of diphosphane. Simultaneously polyphosphorous compounds were formed. Similar reactions were observed for Ti^IV^ compoudns (Baum *et al.* 2004). [Cp_2_TiCl_2_](Cp = C_5_H_5_) reacts with ^t^Bu_2_P—P(Li)—P^t^Bu_2_ or with ^t^Bu_2_P—P(SiMe_3_)Li, respectively yielding Ti(III) complex [Cp_2_Ti{*µ*-Cl)_2_Li(THF)_2_].

(I) crystallizes with one molecule in the asymmetric unit (Fig 1). The iron centre is tetrahedrally coordinated by a chelating amidinate ligand and two chloride atoms. One of them bridges to a lithium ion, which is solvated by two DME molecules. In contrast to the related complexes of iron(II) that includes 2,4-bis(2,6-diisopropylphenylimido) pentane ligand, with both chloride atoms bridging Fe^II^ and Li centres, the presented complex displays one bridging and one terminal chloride atom. The Cl—Fe—Cl angle value in this compound is about 7° wider than analogous one in the iron (II) complexes with 2,4-bis(2,6-diisopropylphenylimido) pentane ligands, and two bridging chloride atoms (Smith *et al.*2001). In comparison with complex [{PhC(N-2,6-^i^Pr_2_C_6_H_3_)_2_}FeCl(*µ*-Cl)Li(THF)_3_] the Cl—N—Cl angle is about 15° smaller (Sciarone *et al.* 2006). The N(1)—Fe(1)—N(2) bond angle is 92.46 (9)°, while the N(1)—Fe(1)—Cl(1), N(1)—Fe(1)—Cl(2), N(2)—Fe(1)—Cl(1), N(2)—Fe(1)—Cl(2) bond angles are - 117.92 (6)°, 114.82 (6)°, 111.77 (6)°, 116.86 (6)°, respectively. The Fe—N and Fe—Cl bond lengths do not differ significantly from typical values (Evans *et al.* 2003).

### Experimental

This work was carried out using the standard vacuum-nitrogen line and Schlenk techiques. [LFeCl_2_] (L = {(2,6-^i^Pr_2_H_3_C_6_)N(CH_3_)C}_2_CH]- and ^t^Bu_2_P—P(SiMe_3_)Li.2DME were prepared according to the procedure in the literature (Panda *et al. *2002; Kovacs *et al.* 1996). Solution of 0.412 g (1.03 mmol) ^t^Bu_2_P—P(SiMe_3_)Li.2THF in 2 ml DME was addded dropwise into solution of 0.303 g (0.515 mmol) [LFeCl_2_] (L = [{2,6-^i^Pr_2_H_3_C_6_)N(CH_3_)C}_2_CH-]) in 2 ml of DME at 243 K. The mixture immediately turned orange. The resultant solution was warmed to room temperature. Then the volume was reduced to about 2 ml and the concentrated solution stored for a few days at 243 K and the solution yielded orange crystals of (I).

### Refinement

All H atoms were fixed geometrically and treated as riding with C—H = 0.95 Å (aromatic), 0.99 Å (methylene), 0.98 Å (methyl) and 1.00 Å (methine) with Uiso(H) = 1.2Ueq ( aromatic, methine, methylene) and Uiso(H) = 1.5Ueq (methyl).

### Crystal data

**Table:**

[FeLi(C_29_H_41_N_2_)Cl_2_(C_4_H_10_O_2_)_2_]	*F*(000) = 1568
*M**_r_* = 731.57	*D*_x_ = 1.212 Mg m^−^^3^
Monoclinic, *P*2_1_/*c*	Mo *K*α radiation, λ = 0.71073 Å
Hall symbol: -P 2ybc	Cell parameters from 13275 reflections
*a* = 10.1467 (5) Å	θ = 2.3–28.8°
*b* = 19.8186 (10) Å	µ = 0.55 mm^−^^1^
*c* = 20.6289 (11) Å	*T* = 150 K
β = 104.962 (4)°	Block, yellow
*V* = 4007.7 (4) Å^3^	0.27 × 0.24 × 0.13 mm
*Z* = 4	

### Data collection

**Table:**

Oxford Diffraction Xcalibur Sapphire2 (large Be window) diffractometer	7045 independent reflections
graphite	4320 reflections with *I* > 2σ(*I*)
Detector resolution: 8.1883 pixels mm^-1^	*R*_int_ = 0.042
ω scans	θ_max_ = 25°, θ_min_ = 2.3°
Absorption correction: multi-scan (*CrysAlis PRO*; Oxford Diffraction, 2009)	*h* = −11→12
*T*_min_ = 0.983, *T*_max_ = 1	*k* = −23→21
23717 measured reflections	*l* = −24→24

### Refinement

**Table:**

Refinement on *F*^2^	Primary atom site location: structure-invariant direct methods
Least-squares matrix: full	Secondary atom site location: difference Fourier map
*R*[*F*^2^ > 2σ(*F*^2^)] = 0.041	Hydrogen site location: inferred from neighbouring sites
*wR*(*F*^2^) = 0.108	H-atom parameters constrained
*S* = 0.89	*w* = 1/[σ^2^(*F*_o_^2^) + (0.0623*P*)^2^] where *P* = (*F*_o_^2^ + 2*F*_c_^2^)/3
7045 reflections	(Δ/σ)_max_ < 0.001
438 parameters	Δρ_max_ = 1.63 e Å^−^^3^
0 restraints	Δρ_min_ = −0.29 e Å^−^^3^

### Special details

**Table:**

Experimental. CrysAlisPro, Oxford Diffraction Ltd., Version 1.171.33.52 (release 06-11-2009 CrysAlis171 .NET) (compiled Nov 6 2009,16:24:50) Empirical absorption correction using spherical harmonics, implemented in SCALE3 ABSPACK scaling algorithm.
Geometry. All esds (except the esd in the dihedral angle between two l.s. planes) are estimated using the full covariance matrix. The cell esds are taken into account individually in the estimation of esds in distances, angles and torsion angles; correlations between esds in cell parameters are only used when they are defined by crystal symmetry. An approximate (isotropic) treatment of cell esds is used for estimating esds involving l.s. planes.
Refinement. Refinement of *F*^2^ against ALL reflections. The weighted *R*-factor wR and goodness of fit *S* are based on *F*^2^, conventional *R*-factors *R* are based on *F*, with *F* set to zero for negative *F*^2^. The threshold expression of *F*^2^ > σ(*F*^2^) is used only for calculating *R*-factors(gt) etc. and is not relevant to the choice of reflections for refinement. *R*-factors based on *F*^2^ are statistically about twice as large as those based on *F*, and *R*- factors based on ALL data will be even larger.

### Fractional atomic coordinates and isotropic or equivalent isotropic displacement parameters (Å^2^)

**Table:**

	*x*	*y*	*z*	*U*_iso_*/*U*_eq_	
Fe1	0.31716 (4)	0.24720 (2)	0.352332 (17)	0.02132 (12)	
Cl1	0.08335 (7)	0.25572 (4)	0.32310 (4)	0.0417 (2)	
Cl2	0.37292 (6)	0.25434 (4)	0.25030 (3)	0.03012 (17)	
O1	0.11678 (19)	0.15479 (10)	0.17693 (9)	0.0342 (5)	
O2	−0.07181 (18)	0.24976 (11)	0.14320 (9)	0.0348 (5)	
O3	0.1702 (2)	0.26965 (10)	0.08043 (9)	0.0332 (5)	
O4	0.13980 (19)	0.36490 (10)	0.16866 (9)	0.0305 (5)	
N1	0.4027 (2)	0.16872 (11)	0.41070 (10)	0.0210 (5)	
N2	0.4048 (2)	0.31567 (11)	0.42353 (10)	0.0214 (5)	
C1	0.4141 (3)	0.10469 (13)	0.37950 (12)	0.0210 (6)	
C2	0.5305 (3)	0.09225 (14)	0.35722 (13)	0.0262 (7)	
C3	0.5429 (3)	0.02954 (15)	0.32907 (13)	0.0319 (7)	
H3A	0.6225	0.0199	0.3146	0.038*	
C4	0.4431 (3)	−0.01886 (15)	0.32163 (13)	0.0323 (7)	
H4A	0.4545	−0.0616	0.3029	0.039*	
C5	0.3266 (3)	−0.00494 (14)	0.34146 (12)	0.0287 (7)	
H5A	0.2571	−0.0382	0.3356	0.034*	
C6	0.3087 (3)	0.05698 (14)	0.37007 (12)	0.0241 (6)	
C7	0.6441 (3)	0.14354 (15)	0.36438 (14)	0.0322 (7)	
H7A	0.6075	0.188	0.3746	0.039*	
C8	0.7677 (3)	0.12627 (19)	0.42361 (15)	0.0479 (9)	
H8A	0.7398	0.1275	0.4657	0.072*	
H8B	0.8404	0.1593	0.4256	0.072*	
H8C	0.8011	0.081	0.4171	0.072*	
C9	0.6902 (3)	0.15230 (17)	0.30007 (15)	0.0401 (8)	
H9A	0.6128	0.1672	0.2638	0.06*	
H9B	0.7245	0.1092	0.2879	0.06*	
H9C	0.7629	0.1862	0.3073	0.06*	
C10	0.1767 (3)	0.06997 (15)	0.38942 (13)	0.0282 (7)	
H10A	0.184	0.1153	0.4115	0.034*	
C11	0.0567 (3)	0.07233 (17)	0.32694 (15)	0.0401 (8)	
H11A	0.0728	0.1076	0.2966	0.06*	
H11B	−0.0273	0.0823	0.3401	0.06*	
H11C	0.0478	0.0286	0.3041	0.06*	
C12	0.1497 (3)	0.01756 (16)	0.43954 (15)	0.0419 (8)	
H12A	0.2275	0.0163	0.4792	0.063*	
H12B	0.1373	−0.0271	0.4183	0.063*	
H12C	0.0671	0.03	0.453	0.063*	
C13	0.4199 (3)	0.38317 (14)	0.40014 (12)	0.0219 (6)	
C14	0.3225 (3)	0.43371 (14)	0.40092 (12)	0.0240 (6)	
C15	0.3387 (3)	0.49617 (14)	0.37277 (12)	0.0271 (7)	
H15A	0.2735	0.5307	0.3722	0.033*	
C16	0.4468 (3)	0.50898 (14)	0.34572 (13)	0.0288 (7)	
H16A	0.4558	0.5519	0.3269	0.035*	
C17	0.5417 (3)	0.45944 (15)	0.34609 (13)	0.0301 (7)	
H17A	0.6166	0.4689	0.3278	0.036*	
C18	0.5309 (3)	0.39550 (14)	0.37261 (13)	0.0257 (7)	
C19	0.2041 (3)	0.42393 (15)	0.43281 (13)	0.0309 (7)	
H19A	0.2125	0.378	0.4536	0.037*	
C20	0.2098 (3)	0.47611 (17)	0.48840 (15)	0.0435 (8)	
H20A	0.2999	0.4747	0.5204	0.065*	
H20B	0.1395	0.4657	0.5117	0.065*	
H20C	0.1937	0.5213	0.4686	0.065*	
C21	0.0649 (3)	0.42854 (16)	0.38159 (15)	0.0405 (8)	
H21A	0.0539	0.3903	0.3506	0.061*	
H21B	0.0594	0.4708	0.3563	0.061*	
H21C	−0.0076	0.4275	0.4051	0.061*	
C22	0.6375 (3)	0.34182 (15)	0.37306 (14)	0.0311 (7)	
H22A	0.5903	0.2972	0.3694	0.037*	
C23	0.7495 (3)	0.34164 (19)	0.43980 (16)	0.0496 (9)	
H23A	0.7085	0.3318	0.4769	0.074*	
H23B	0.7937	0.386	0.4468	0.074*	
H23C	0.8176	0.307	0.4381	0.074*	
C24	0.7036 (3)	0.34645 (17)	0.31492 (15)	0.0409 (8)	
H24A	0.6332	0.3427	0.2724	0.061*	
H24B	0.7697	0.3097	0.3182	0.061*	
H24C	0.7504	0.3899	0.3166	0.061*	
C25	0.4912 (3)	0.11037 (15)	0.51887 (13)	0.0319 (7)	
H25A	0.5607	0.0865	0.5023	0.048*	
H25B	0.4121	0.0809	0.5152	0.048*	
H25C	0.5292	0.1229	0.566	0.048*	
C26	0.4476 (3)	0.17321 (14)	0.47756 (12)	0.0231 (6)	
C27	0.4599 (3)	0.23406 (13)	0.51291 (13)	0.0251 (7)	
H27A	0.4778	0.23	0.5603	0.03*	
C28	0.4495 (3)	0.30037 (14)	0.48817 (13)	0.0247 (6)	
C29	0.4927 (3)	0.35557 (15)	0.54001 (13)	0.0349 (8)	
H29A	0.5307	0.3935	0.5203	0.052*	
H29B	0.562	0.338	0.5786	0.052*	
H29C	0.4133	0.371	0.5548	0.052*	
C30	0.2147 (3)	0.10323 (17)	0.17719 (16)	0.0470 (9)	
H30A	0.2012	0.0661	0.2062	0.07*	
H30B	0.3068	0.1217	0.1941	0.07*	
H30C	0.2035	0.0864	0.1314	0.07*	
C31	−0.0196 (3)	0.13365 (17)	0.14901 (16)	0.0451 (9)	
H31A	−0.0364	0.0899	0.1686	0.054*	
H31B	−0.037	0.1282	0.0998	0.054*	
C32	−0.1108 (3)	0.18644 (18)	0.16482 (16)	0.0434 (9)	
H32A	−0.207	0.1764	0.1414	0.052*	
H32B	−0.1024	0.1875	0.2137	0.052*	
C33	−0.1580 (3)	0.3032 (2)	0.15367 (19)	0.0601 (11)	
H33A	−0.2521	0.294	0.1283	0.09*	
H33B	−0.1273	0.3458	0.1384	0.09*	
H33C	−0.1538	0.3064	0.2016	0.09*	
C34	0.1146 (4)	0.22442 (18)	0.02709 (14)	0.0482 (9)	
H34A	0.1458	0.2371	−0.0124	0.072*	
H34B	0.0147	0.2265	0.0161	0.072*	
H34C	0.1447	0.1784	0.0407	0.072*	
C35	0.1480 (3)	0.33721 (17)	0.05910 (13)	0.0404 (8)	
H35A	0.0491	0.3461	0.0418	0.049*	
H35B	0.193	0.3465	0.0228	0.049*	
C36	0.2071 (3)	0.38119 (16)	0.11862 (14)	0.0392 (8)	
H36A	0.3063	0.3729	0.1354	0.047*	
H36B	0.193	0.4294	0.1061	0.047*	
C37	0.1880 (3)	0.40560 (16)	0.22734 (13)	0.0398 (8)	
H37A	0.1656	0.453	0.2162	0.06*	
H37B	0.2871	0.4006	0.2439	0.06*	
H37C	0.1443	0.391	0.2622	0.06*	
Li1	0.1402 (4)	0.2579 (2)	0.1758 (2)	0.0293 (11)	

### Atomic displacement parameters (Å^2^)

**Table:**

	*U*^11^	*U*^22^	*U*^33^	*U*^12^	*U*^13^	*U*^23^
Fe1	0.0233 (2)	0.0180 (2)	0.01979 (19)	0.00204 (18)	0.00035 (15)	−0.00038 (17)
Cl1	0.0236 (3)	0.0342 (5)	0.0597 (5)	0.0032 (3)	−0.0029 (3)	−0.0087 (4)
Cl2	0.0277 (3)	0.0388 (4)	0.0222 (3)	0.0011 (3)	0.0036 (3)	0.0002 (3)
O1	0.0339 (12)	0.0252 (12)	0.0404 (11)	−0.0029 (9)	0.0037 (9)	−0.0042 (9)
O2	0.0246 (10)	0.0364 (13)	0.0423 (11)	−0.0010 (10)	0.0063 (9)	0.0057 (10)
O3	0.0381 (12)	0.0342 (13)	0.0264 (10)	−0.0035 (9)	0.0066 (9)	−0.0030 (9)
O4	0.0361 (12)	0.0285 (12)	0.0263 (10)	−0.0036 (9)	0.0068 (9)	0.0003 (9)
N1	0.0207 (12)	0.0199 (13)	0.0226 (11)	0.0009 (10)	0.0061 (10)	0.0016 (10)
N2	0.0215 (12)	0.0190 (13)	0.0223 (12)	0.0006 (10)	0.0030 (10)	0.0005 (9)
C1	0.0267 (15)	0.0162 (15)	0.0198 (13)	0.0059 (12)	0.0057 (12)	0.0044 (11)
C2	0.0264 (15)	0.0233 (17)	0.0298 (15)	0.0071 (12)	0.0093 (13)	0.0056 (12)
C3	0.0350 (17)	0.0284 (18)	0.0384 (16)	0.0116 (14)	0.0203 (14)	0.0045 (14)
C4	0.0454 (18)	0.0209 (17)	0.0331 (16)	0.0073 (14)	0.0146 (14)	−0.0019 (13)
C5	0.0370 (17)	0.0200 (16)	0.0290 (15)	0.0003 (13)	0.0087 (14)	0.0015 (12)
C6	0.0284 (15)	0.0197 (16)	0.0249 (14)	0.0046 (12)	0.0086 (12)	0.0048 (11)
C7	0.0265 (16)	0.0265 (18)	0.0480 (18)	0.0058 (13)	0.0175 (14)	0.0002 (14)
C8	0.0365 (19)	0.063 (3)	0.0453 (19)	−0.0069 (17)	0.0127 (16)	−0.0029 (17)
C9	0.0356 (18)	0.035 (2)	0.0545 (19)	0.0038 (15)	0.0204 (16)	0.0110 (16)
C10	0.0305 (16)	0.0188 (16)	0.0383 (16)	−0.0012 (12)	0.0143 (14)	−0.0015 (12)
C11	0.0309 (17)	0.039 (2)	0.0504 (19)	0.0037 (15)	0.0106 (15)	−0.0020 (16)
C12	0.0468 (19)	0.035 (2)	0.053 (2)	−0.0013 (16)	0.0291 (17)	0.0045 (15)
C13	0.0240 (15)	0.0196 (16)	0.0180 (13)	−0.0034 (12)	−0.0021 (12)	−0.0032 (11)
C14	0.0265 (15)	0.0195 (16)	0.0235 (14)	−0.0029 (12)	0.0018 (12)	−0.0040 (11)
C15	0.0297 (16)	0.0188 (16)	0.0286 (15)	0.0026 (12)	0.0000 (13)	−0.0024 (12)
C16	0.0375 (17)	0.0163 (16)	0.0309 (15)	−0.0054 (13)	0.0060 (14)	−0.0021 (12)
C17	0.0300 (16)	0.0264 (18)	0.0337 (16)	−0.0085 (13)	0.0079 (13)	−0.0045 (13)
C18	0.0233 (15)	0.0218 (16)	0.0275 (14)	−0.0034 (12)	−0.0017 (12)	−0.0044 (12)
C19	0.0394 (17)	0.0197 (17)	0.0371 (16)	0.0074 (13)	0.0161 (14)	0.0057 (13)
C20	0.057 (2)	0.038 (2)	0.0402 (18)	0.0106 (17)	0.0197 (16)	0.0022 (15)
C21	0.0349 (18)	0.030 (2)	0.060 (2)	0.0003 (15)	0.0194 (16)	−0.0050 (16)
C22	0.0225 (15)	0.0242 (17)	0.0440 (17)	0.0002 (13)	0.0036 (14)	−0.0008 (14)
C23	0.0268 (17)	0.060 (3)	0.055 (2)	0.0082 (16)	−0.0013 (16)	0.0030 (18)
C24	0.0328 (18)	0.037 (2)	0.0567 (19)	0.0038 (15)	0.0182 (16)	−0.0017 (16)
C25	0.0375 (17)	0.0300 (18)	0.0276 (15)	0.0082 (14)	0.0073 (13)	0.0085 (13)
C26	0.0216 (15)	0.0248 (16)	0.0229 (14)	0.0037 (12)	0.0059 (12)	0.0050 (12)
C27	0.0302 (15)	0.0270 (18)	0.0173 (13)	0.0053 (12)	0.0048 (11)	0.0022 (11)
C28	0.0202 (15)	0.0265 (17)	0.0251 (14)	0.0036 (12)	0.0014 (12)	−0.0028 (12)
C29	0.0442 (19)	0.0298 (19)	0.0243 (15)	0.0045 (14)	−0.0026 (14)	−0.0046 (13)
C30	0.061 (2)	0.033 (2)	0.0482 (19)	0.0127 (17)	0.0172 (17)	−0.0055 (16)
C31	0.045 (2)	0.037 (2)	0.0465 (19)	−0.0140 (16)	−0.0005 (16)	−0.0045 (16)
C32	0.0274 (17)	0.049 (2)	0.0492 (19)	−0.0150 (16)	0.0012 (15)	0.0062 (17)
C33	0.037 (2)	0.057 (3)	0.087 (3)	0.0160 (19)	0.016 (2)	0.009 (2)
C34	0.062 (2)	0.049 (2)	0.0341 (18)	−0.0061 (18)	0.0130 (17)	−0.0162 (16)
C35	0.050 (2)	0.043 (2)	0.0288 (16)	−0.0042 (16)	0.0110 (15)	0.0047 (15)
C36	0.052 (2)	0.033 (2)	0.0356 (17)	−0.0105 (16)	0.0160 (15)	0.0028 (14)
C37	0.058 (2)	0.0288 (19)	0.0306 (16)	−0.0031 (16)	0.0079 (15)	−0.0096 (14)
Li1	0.027 (2)	0.023 (3)	0.035 (2)	−0.004 (2)	0.004 (2)	−0.004 (2)

### Geometric parameters (Å, °)

**Table:**

Fe1—N1	2.020 (2)	C16—C17	1.373 (4)
Fe1—N2	2.029 (2)	C16—H16A	0.95
Fe1—Cl1	2.2982 (8)	C17—C18	1.396 (4)
Fe1—Cl2	2.3207 (7)	C17—H17A	0.95
Cl2—Li1	2.463 (4)	C18—C22	1.516 (4)
O1—C31	1.418 (3)	C19—C21	1.533 (4)
O1—C30	1.424 (4)	C19—C20	1.534 (4)
O1—Li1	2.058 (5)	C19—H19A	1
O2—C32	1.422 (4)	C20—H20A	0.98
O2—C33	1.424 (4)	C20—H20B	0.98
O2—Li1	2.088 (5)	C20—H20C	0.98
O3—C35	1.409 (4)	C21—H21A	0.98
O3—C34	1.419 (3)	C21—H21B	0.98
O3—Li1	2.081 (5)	C21—H21C	0.98
O4—C36	1.415 (3)	C22—C24	1.521 (4)
O4—C37	1.432 (3)	C22—C23	1.543 (4)
O4—Li1	2.126 (5)	C22—H22A	1
N1—C26	1.339 (3)	C23—H23A	0.98
N1—C1	1.441 (3)	C23—H23B	0.98
N2—C28	1.328 (3)	C23—H23C	0.98
N2—C13	1.443 (3)	C24—H24A	0.98
C1—C2	1.395 (4)	C24—H24B	0.98
C1—C6	1.403 (4)	C24—H24C	0.98
C2—C3	1.391 (4)	C25—C26	1.510 (4)
C2—C7	1.515 (4)	C25—H25A	0.98
C3—C4	1.375 (4)	C25—H25B	0.98
C3—H3A	0.95	C25—H25C	0.98
C4—C5	1.374 (4)	C26—C27	1.398 (4)
C4—H4A	0.95	C27—C28	1.404 (4)
C5—C6	1.394 (4)	C27—H27A	0.95
C5—H5A	0.95	C28—C29	1.513 (4)
C6—C10	1.515 (4)	C29—H29A	0.98
C7—C9	1.526 (4)	C29—H29B	0.98
C7—C8	1.546 (4)	C29—H29C	0.98
C7—H7A	1	C30—H30A	0.98
C8—H8A	0.98	C30—H30B	0.98
C8—H8B	0.98	C30—H30C	0.98
C8—H8C	0.98	C31—C32	1.488 (5)
C9—H9A	0.98	C31—H31A	0.99
C9—H9B	0.98	C31—H31B	0.99
C9—H9C	0.98	C32—H32A	0.99
C10—C11	1.528 (4)	C32—H32B	0.99
C10—C12	1.539 (4)	C33—H33A	0.98
C10—H10A	1	C33—H33B	0.98
C11—H11A	0.98	C33—H33C	0.98
C11—H11B	0.98	C34—H34A	0.98
C11—H11C	0.98	C34—H34B	0.98
C12—H12A	0.98	C34—H34C	0.98
C12—H12B	0.98	C35—C36	1.499 (4)
C12—H12C	0.98	C35—H35A	0.99
C13—C18	1.407 (4)	C35—H35B	0.99
C13—C14	1.410 (4)	C36—H36A	0.99
C14—C15	1.395 (4)	C36—H36B	0.99
C14—C19	1.524 (4)	C37—H37A	0.98
C15—C16	1.377 (4)	C37—H37B	0.98
C15—H15A	0.95	C37—H37C	0.98
			
N1—Fe1—N2	92.46 (9)	C19—C20—H20C	109.5
N1—Fe1—Cl1	117.92 (6)	H20A—C20—H20C	109.5
N2—Fe1—Cl1	111.77 (6)	H20B—C20—H20C	109.5
N1—Fe1—Cl2	114.82 (6)	C19—C21—H21A	109.5
N2—Fe1—Cl2	116.86 (6)	C19—C21—H21B	109.5
Cl1—Fe1—Cl2	103.56 (3)	H21A—C21—H21B	109.5
Fe1—Cl2—Li1	98.54 (11)	C19—C21—H21C	109.5
C31—O1—C30	113.3 (2)	H21A—C21—H21C	109.5
C31—O1—Li1	113.1 (2)	H21B—C21—H21C	109.5
C30—O1—Li1	129.1 (2)	C18—C22—C24	114.3 (2)
C32—O2—C33	112.1 (3)	C18—C22—C23	111.6 (2)
C32—O2—Li1	108.3 (2)	C24—C22—C23	109.3 (2)
C33—O2—Li1	121.0 (2)	C18—C22—H22A	107.1
C35—O3—C34	111.0 (2)	C24—C22—H22A	107.1
C35—O3—Li1	110.6 (2)	C23—C22—H22A	107.1
C34—O3—Li1	122.5 (2)	C22—C23—H23A	109.5
C36—O4—C37	111.4 (2)	C22—C23—H23B	109.5
C36—O4—Li1	106.6 (2)	H23A—C23—H23B	109.5
C37—O4—Li1	120.6 (2)	C22—C23—H23C	109.5
C26—N1—C1	118.2 (2)	H23A—C23—H23C	109.5
C26—N1—Fe1	122.84 (18)	H23B—C23—H23C	109.5
C1—N1—Fe1	118.91 (15)	C22—C24—H24A	109.5
C28—N2—C13	120.6 (2)	C22—C24—H24B	109.5
C28—N2—Fe1	123.12 (19)	H24A—C24—H24B	109.5
C13—N2—Fe1	116.26 (15)	C22—C24—H24C	109.5
C2—C1—C6	121.0 (2)	H24A—C24—H24C	109.5
C2—C1—N1	118.4 (2)	H24B—C24—H24C	109.5
C6—C1—N1	120.6 (2)	C26—C25—H25A	109.5
C3—C2—C1	118.1 (3)	C26—C25—H25B	109.5
C3—C2—C7	119.4 (3)	H25A—C25—H25B	109.5
C1—C2—C7	122.4 (3)	C26—C25—H25C	109.5
C4—C3—C2	121.8 (3)	H25A—C25—H25C	109.5
C4—C3—H3A	119.1	H25B—C25—H25C	109.5
C2—C3—H3A	119.1	N1—C26—C27	123.7 (2)
C5—C4—C3	119.5 (3)	N1—C26—C25	119.9 (2)
C5—C4—H4A	120.3	C27—C26—C25	116.3 (2)
C3—C4—H4A	120.3	C26—C27—C28	129.0 (2)
C4—C5—C6	121.3 (3)	C26—C27—H27A	115.5
C4—C5—H5A	119.4	C28—C27—H27A	115.5
C6—C5—H5A	119.4	N2—C28—C27	123.6 (2)
C5—C6—C1	118.3 (2)	N2—C28—C29	120.4 (2)
C5—C6—C10	118.8 (3)	C27—C28—C29	116.0 (2)
C1—C6—C10	122.9 (2)	C28—C29—H29A	109.5
C2—C7—C9	112.8 (2)	C28—C29—H29B	109.5
C2—C7—C8	111.7 (2)	H29A—C29—H29B	109.5
C9—C7—C8	110.1 (2)	C28—C29—H29C	109.5
C2—C7—H7A	107.3	H29A—C29—H29C	109.5
C9—C7—H7A	107.3	H29B—C29—H29C	109.5
C8—C7—H7A	107.3	O1—C30—H30A	109.5
C7—C8—H8A	109.5	O1—C30—H30B	109.5
C7—C8—H8B	109.5	H30A—C30—H30B	109.5
H8A—C8—H8B	109.5	O1—C30—H30C	109.5
C7—C8—H8C	109.5	H30A—C30—H30C	109.5
H8A—C8—H8C	109.5	H30B—C30—H30C	109.5
H8B—C8—H8C	109.5	O1—C31—C32	107.5 (2)
C7—C9—H9A	109.5	O1—C31—H31A	110.2
C7—C9—H9B	109.5	C32—C31—H31A	110.2
H9A—C9—H9B	109.5	O1—C31—H31B	110.2
C7—C9—H9C	109.5	C32—C31—H31B	110.2
H9A—C9—H9C	109.5	H31A—C31—H31B	108.5
H9B—C9—H9C	109.5	O2—C32—C31	108.2 (3)
C6—C10—C11	110.4 (2)	O2—C32—H32A	110.1
C6—C10—C12	112.7 (2)	C31—C32—H32A	110.1
C11—C10—C12	110.2 (2)	O2—C32—H32B	110.1
C6—C10—H10A	107.8	C31—C32—H32B	110.1
C11—C10—H10A	107.8	H32A—C32—H32B	108.4
C12—C10—H10A	107.8	O2—C33—H33A	109.5
C10—C11—H11A	109.5	O2—C33—H33B	109.5
C10—C11—H11B	109.5	H33A—C33—H33B	109.5
H11A—C11—H11B	109.5	O2—C33—H33C	109.5
C10—C11—H11C	109.5	H33A—C33—H33C	109.5
H11A—C11—H11C	109.5	H33B—C33—H33C	109.5
H11B—C11—H11C	109.5	O3—C34—H34A	109.5
C10—C12—H12A	109.5	O3—C34—H34B	109.5
C10—C12—H12B	109.5	H34A—C34—H34B	109.5
H12A—C12—H12B	109.5	O3—C34—H34C	109.5
C10—C12—H12C	109.5	H34A—C34—H34C	109.5
H12A—C12—H12C	109.5	H34B—C34—H34C	109.5
H12B—C12—H12C	109.5	O3—C35—C36	107.4 (2)
C18—C13—C14	121.1 (3)	O3—C35—H35A	110.2
C18—C13—N2	117.4 (2)	C36—C35—H35A	110.2
C14—C13—N2	121.3 (2)	O3—C35—H35B	110.2
C15—C14—C13	117.9 (3)	C36—C35—H35B	110.2
C15—C14—C19	118.9 (3)	H35A—C35—H35B	108.5
C13—C14—C19	123.2 (2)	O4—C36—C35	107.4 (2)
C16—C15—C14	121.6 (3)	O4—C36—H36A	110.2
C16—C15—H15A	119.2	C35—C36—H36A	110.2
C14—C15—H15A	119.2	O4—C36—H36B	110.2
C17—C16—C15	119.8 (3)	C35—C36—H36B	110.2
C17—C16—H16A	120.1	H36A—C36—H36B	108.5
C15—C16—H16A	120.1	O4—C37—H37A	109.5
C16—C17—C18	121.7 (3)	O4—C37—H37B	109.5
C16—C17—H17A	119.2	H37A—C37—H37B	109.5
C18—C17—H17A	119.2	O4—C37—H37C	109.5
C17—C18—C13	117.9 (3)	H37A—C37—H37C	109.5
C17—C18—C22	120.8 (3)	H37B—C37—H37C	109.5
C13—C18—C22	121.3 (3)	O1—Li1—O3	99.6 (2)
C14—C19—C21	112.6 (2)	O1—Li1—O2	79.33 (18)
C14—C19—C20	110.8 (2)	O3—Li1—O2	95.40 (19)
C21—C19—C20	109.0 (2)	O1—Li1—O4	173.3 (3)
C14—C19—H19A	108.1	O3—Li1—O4	79.66 (18)
C21—C19—H19A	108.1	O2—Li1—O4	94.1 (2)
C20—C19—H19A	108.1	O1—Li1—Cl2	93.41 (18)
C19—C20—H20A	109.5	O3—Li1—Cl2	104.02 (19)
C19—C20—H20B	109.5	O2—Li1—Cl2	160.2 (2)
H20A—C20—H20B	109.5	O4—Li1—Cl2	93.21 (17)
			
N1—Fe1—Cl2—Li1	125.89 (13)	C15—C14—C19—C20	−57.1 (3)
N2—Fe1—Cl2—Li1	−127.41 (13)	C13—C14—C19—C20	120.9 (3)
Cl1—Fe1—Cl2—Li1	−4.06 (12)	C17—C18—C22—C24	−31.1 (4)
N2—Fe1—N1—C26	21.2 (2)	C13—C18—C22—C24	150.4 (3)
Cl1—Fe1—N1—C26	−95.1 (2)	C17—C18—C22—C23	93.5 (3)
Cl2—Fe1—N1—C26	142.44 (18)	C13—C18—C22—C23	−85.0 (3)
N2—Fe1—N1—C1	−159.79 (19)	C1—N1—C26—C27	170.7 (2)
Cl1—Fe1—N1—C1	83.93 (19)	Fe1—N1—C26—C27	−10.3 (4)
Cl2—Fe1—N1—C1	−38.6 (2)	C1—N1—C26—C25	−8.0 (4)
N1—Fe1—N2—C28	−21.1 (2)	Fe1—N1—C26—C25	171.04 (19)
Cl1—Fe1—N2—C28	100.3 (2)	N1—C26—C27—C28	−10.4 (5)
Cl2—Fe1—N2—C28	−140.67 (18)	C25—C26—C27—C28	168.3 (3)
N1—Fe1—N2—C13	157.42 (18)	C13—N2—C28—C27	−168.6 (3)
Cl1—Fe1—N2—C13	−81.13 (18)	Fe1—N2—C28—C27	9.9 (4)
Cl2—Fe1—N2—C13	37.9 (2)	C13—N2—C28—C29	11.5 (4)
C26—N1—C1—C2	−92.5 (3)	Fe1—N2—C28—C29	−170.0 (2)
Fe1—N1—C1—C2	88.5 (2)	C26—C27—C28—N2	10.6 (5)
C26—N1—C1—C6	89.4 (3)	C26—C27—C28—C29	−169.5 (3)
Fe1—N1—C1—C6	−89.7 (2)	C30—O1—C31—C32	168.3 (2)
C6—C1—C2—C3	−3.9 (4)	Li1—O1—C31—C32	−33.3 (3)
N1—C1—C2—C3	178.0 (2)	C33—O2—C32—C31	177.4 (2)
C6—C1—C2—C7	177.8 (2)	Li1—O2—C32—C31	−46.5 (3)
N1—C1—C2—C7	−0.3 (4)	O1—C31—C32—O2	52.7 (3)
C1—C2—C3—C4	1.4 (4)	C34—O3—C35—C36	178.8 (2)
C7—C2—C3—C4	179.8 (2)	Li1—O3—C35—C36	39.5 (3)
C2—C3—C4—C5	1.0 (4)	C37—O4—C36—C35	−178.9 (2)
C3—C4—C5—C6	−1.1 (4)	Li1—O4—C36—C35	47.7 (3)
C4—C5—C6—C1	−1.3 (4)	O3—C35—C36—O4	−59.3 (3)
C4—C5—C6—C10	178.6 (2)	C31—O1—Li1—O3	−86.8 (2)
C2—C1—C6—C5	3.8 (4)	C30—O1—Li1—O3	67.4 (3)
N1—C1—C6—C5	−178.1 (2)	C31—O1—Li1—O2	6.9 (2)
C2—C1—C6—C10	−176.0 (2)	C30—O1—Li1—O2	161.2 (2)
N1—C1—C6—C10	2.0 (4)	C31—O1—Li1—Cl2	168.4 (2)
C3—C2—C7—C9	48.4 (3)	C30—O1—Li1—Cl2	−37.4 (3)
C1—C2—C7—C9	−133.3 (3)	C35—O3—Li1—O1	162.1 (2)
C3—C2—C7—C8	−76.3 (3)	C34—O3—Li1—O1	28.3 (3)
C1—C2—C7—C8	102.0 (3)	C35—O3—Li1—O2	82.1 (2)
C5—C6—C10—C11	−65.9 (3)	C34—O3—Li1—O2	−51.8 (3)
C1—C6—C10—C11	114.0 (3)	C35—O3—Li1—O4	−11.1 (2)
C5—C6—C10—C12	57.9 (3)	C34—O3—Li1—O4	−145.0 (2)
C1—C6—C10—C12	−122.3 (3)	C35—O3—Li1—Cl2	−101.9 (2)
C28—N2—C13—C18	98.2 (3)	C34—O3—Li1—Cl2	124.3 (2)
Fe1—N2—C13—C18	−80.4 (2)	C32—O2—Li1—O1	22.2 (2)
C28—N2—C13—C14	−85.7 (3)	C33—O2—Li1—O1	153.6 (2)
Fe1—N2—C13—C14	95.7 (2)	C32—O2—Li1—O3	121.0 (2)
C18—C13—C14—C15	0.8 (4)	C33—O2—Li1—O3	−107.5 (3)
N2—C13—C14—C15	−175.2 (2)	C32—O2—Li1—O4	−159.0 (2)
C18—C13—C14—C19	−177.3 (2)	C33—O2—Li1—O4	−27.6 (3)
N2—C13—C14—C19	6.8 (4)	C32—O2—Li1—Cl2	−47.7 (8)
C13—C14—C15—C16	−0.8 (4)	C33—O2—Li1—Cl2	83.8 (8)
C19—C14—C15—C16	177.3 (2)	C36—O4—Li1—O3	−20.9 (2)
C14—C15—C16—C17	0.1 (4)	C37—O4—Li1—O3	−149.1 (2)
C15—C16—C17—C18	0.8 (4)	C36—O4—Li1—O2	−115.6 (2)
C16—C17—C18—C13	−0.9 (4)	C37—O4—Li1—O2	116.2 (2)
C16—C17—C18—C22	−179.4 (2)	C36—O4—Li1—Cl2	82.8 (2)
C14—C13—C18—C17	0.1 (4)	C37—O4—Li1—Cl2	−45.4 (3)
N2—C13—C18—C17	176.1 (2)	Fe1—Cl2—Li1—O1	−82.12 (15)
C14—C13—C18—C22	178.6 (2)	Fe1—Cl2—Li1—O3	177.06 (16)
N2—C13—C18—C22	−5.3 (3)	Fe1—Cl2—Li1—O2	−14.6 (7)
C15—C14—C19—C21	65.2 (3)	Fe1—Cl2—Li1—O4	96.92 (14)
C13—C14—C19—C21	−116.8 (3)		
